# I Am as Incompetent as the Prototypical Group Member: An Investigation of Naturally Occurring Golem Effects in Work Groups

**DOI:** 10.3389/fpsyg.2018.01581

**Published:** 2018-09-11

**Authors:** Alex Leung, Thomas Sy

**Affiliations:** Leadership and Group Dynamics Lab, Department of Psychology, University of California, Riverside, Riverside, CA, United States

**Keywords:** golem effects, implicit followership theories, self-fulfilling prophecy, social identity, performance

## Abstract

Over four decades, research has demonstrated Pygmalion and Galatea effects (positive expectations leading to high performance) across various settings. In contrast, research on the parallel notion of Golem effects (negative expectations leading to low performance) has been largely overlooked. This study is the first to examine the relationship between group-level Implicit Followership Theories (GIFTs) and naturally occurring Golem effects. Integrating the literature on Implicit Followership Theories, self-fulfilling prophecies, and social identity, we propose that negative GIFTs can serve as proxies of expectations for followers that trigger Golem effects in workgroups. Data from 202 followers and 101 leaders provide support for our hypothesized multi-level model, revealing a top-down relationship between negative GIFTs and follower performance through their self-efficacy and effort. Findings highlight the importance of GIFTs in the Golem process, showing that followers’ cognitions and behaviors are shaped by the group’s prototypical attributes. Suggestions for future research are offered, including interpersonal Golem effects, negative GIFTs and negative outcomes, and influence of organizational culture.

## Introduction

For over five decades, research has shown the impact of individuals’ expectations on organizational outcomes, such as work performance (e.g., Eden and Zuk, 1995; Whiteley et al., 2012). Since Rosenthal and Jacobson’s (1968) first demonstration of expectancy effects in which individuals’ positive expectations result in high performance, hundreds of studies have shown the effect of this powerful mechanism across different settings, such as education, military, and industry (McNatt, 2000). In particular, researchers have shed light on the same concept in the workplace and found that leaders’ positive expectations of their followers result in greater follower performance (e.g., Eden and Ravid, 1982; Whiteley et al., 2012). Similarly, research has found that employees with more positive self-expectations tend to perform at a higher level (e.g., Eden and Zuk, 1995; McNatt and Judge, 2004). These findings suggest that expectations play an enormous role in individuals’ work performance. However, expectations may not always result in positive consequences. While positive expectations may promote individuals’ performance, negative expectations, on the flip side, may hamper performance (Babad et al., 1982). In the past, researchers have primarily focused on positive expectancy effects (i.e., Pygmalion and Galatea effects) (e.g., Rosenthal and Jacobson, 1968; Eden and Zuk, 1995) and studies have yet to examine the consequences of the dark variant — Golem effects—particularly in work settings. Golem effects are a special case of self-fulfilling prophecies in which individuals’ negative expectation diminishes their overall performance (Babad et al., 1982). To date, research examining Golem effects is scant, perhaps due to ethical concerns of inducing negative expectancies that may have detrimental outcomes for participants beyond the confines of the study (Oz and Eden, 1994).

The few studies that have investigated Golem effects have done so via indirect means. For example, Oz and Eden (1994) studied Golem effects by manipulating squad leaders’ interpretation of low physical examination scores to counter the natural formation of negative expectations for low performing paratroopers. Specifically, squad leaders were informed that the Bar-Or test (i.e., physical examination) along with past experience in other units do not predict future performance, and those with low Bar-Or scores often perform just as well as individuals with high scores. In the control group, the researcher only described the study and no information was provided regarding predictions of future performance. Results showed that Golem effects may be restrained by changing squad leaders’ interpretations of the low Bar-Or test scores. In particular, participants in the experimental group made substantial improvements on their Bar-Or test (Golem effects restrained), whereas Golem effects were retained for individuals in the control group as indicated by their persistently lower performance. In short, Oz and Eden (1994) indirectly studied Golem effects by showing that low performing individuals (control group) performed worse than an equivalent peer (experimental) group whose leaders’ low expectations for them were mitigated (a process they labeled de-golemization).

More recently, research on Golem effects was investigated directly in an educational setting by inducing supporting instructors with negative expectations in a laboratory study with undergraduate participants (Reynolds, 2007). Specifically, supporting instructors were told that students were put into different conditions based on the result of a management-acumen test, though neither the students nor supporting instructors were aware of the random assignment. Upon dividing the students randomly into three groups (i.e., positive, negative, and control condition), the supporting instructors received different information regarding the students in each group. First, one support instructor was told that she had been assigned the high-performing group and that this group of students would likely perform well on subsequent tests. The second support instructor was informed that she had been given a group with low performance and that these students may perform equally poor in the subsequent assessments. Lastly, the third support instructor was given no information about the students. Results confirmed the linkage between expectations (i.e., positive and negative) and task performance. Specifically, the change in pretest and post-test scores showed that positive expectation led to higher levels of performance, whereas negative expectation led to lower levels of performance. The latter finding reflects the ethical concerns of researchers. The potential damage that may be inflicted by artificially inducing lower expectations has deterred researchers from studying Golem effects for nearly half a century. As such, little is known about Golem effects, particularly in work settings. To date, we are not aware of any field study investigating Golem effects at work.

**FIGURE 1:**
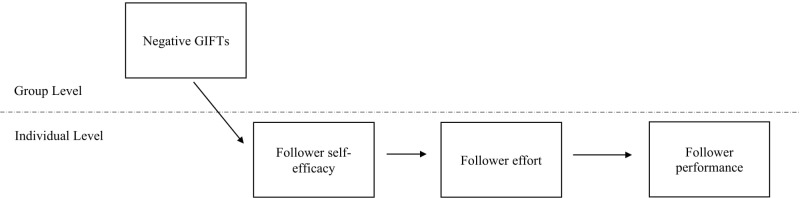
Proposedmulti-level model of negative GIFTs.

One solution that circumvents these ethical concerns and affords investigations into Golem effects is to study it in its natural form (Oz and Eden, 1994). Most research on expectancy effects involves the artificial manipulation of leaders’ expectations for their followers. However, leaders’ expectations for their followers in most work settings occur naturally, without experimental manipulation (Eden, 1990). Accordingly, we refer to *naturally occurring* Golem effects as negative expectancy effects that occur without any form of artificial manipulation (Whiteley et al., 2012). Recent developments on implicit followership theories (IFTs) or conceptions of followers (Sy, 2010) offers a new avenue to investigate *naturally occurring* Golem effects because conceptions of followers can serve as proxies of expectations for followers that trigger Golem effects. In the current study, we investigate naturally occurring Golem effects in organizational settings via IFTs. IFTs exist at both the individual and group levels (Epitropaki et al., 2013). Our focus is on group’s Implicit Followership Theories (GIFTs), specifically, the *Incompetency* schema—due to its direct relevance to performance. Consistent with recent research (Whiteley et al., 2012), we propose that negative schemas of GIFTs (i.e., incompetence) may serve as proxies for performance expectations that trigger naturally occurring Golem effects. Specifically, we propose that GIFTs are associated with individuals’ performance via self-efficacy and effort (see **Figure 1**).

We contribute to the organizational literature in several ways. This study is the first to investigate the relationship between group Implicit Followership Theories (GIFTs) and naturally occurring Golem effects. Our study shows how negative GIFTs may serve as negative in-group expectations for followers which trigger the process of naturally occurring Golem effects, hampering their performance at work. Furthermore, GIFTs provide a new avenue for investigating self-fulfilling prophecies “in the wild” as organizational researchers studying Golem effects are often restricted by the feasibilities of naturalistic organizational settings (e.g., ethical concerns with artificially inducing negative states). We also advance the field of implicit theories by showing the relevance of IFTs in shaping employee outcomes. This insight is particularly important given the decades-long criticism that implicit theories have failed to demonstrate its practical relevance for workplace outcomes (Epitropaki et al., 2013). Finally, we integrate three distinct fields of Implicit Theories, Self-fulfilling Prophecies, and Social Identity to explain how Golem effects may form naturally, shaping followers’ cognitions and behaviors negatively in everyday work settings.

### Group Level Implicit Followership Theories

Over decades, researchers have investigated individuals’ conceptions of leaders, namely implicit leadership theories (e.g., Lord et al., 1984; Offermann et al., 1994). In comparison, researchers have only begun examining the parallel notion of IFTs recently (IFTs; Sy, 2010; van Gils et al., 2010; Whiteley et al., 2012; Tee et al., 2013; Steffens et al., 2016; Wang and Peng, 2016; Alipour et al., 2017).

Whereas IFTs represent conceptions of followers at the individual level (Sy, 2010), GIFTs represent parallel conceptions of followers at the group level. These conceptions are formed at an early age through socialization (Hunt et al., 1990; Antonakis and Dalgas, 2009) and continue to be developed based on interactions with others (Lord and Maher, 1991; Sy, 2010), such as others within their workgroup. GIFTs are represented in the form of prototypes (Rosch and Lloyd, 1978; Sy, 2010), which may represent ideal (i.e., how followers should be), or typical (i.e., how followers typically are) forms. GIFTs are represented by six dimensions (Sy, 2010): Industry, Good Citizen, Enthusiasm, Incompetence, Insubordination, and Conformity. These six dimensions also represent an overall positive follower prototype (Industry, Good Citizen, Enthusiasm) and an overall negative follower prototype (Incompetence, Insubordination, Conformity) (Sy, 2010). Individuals may use GIFTs as a “sense-making” function (Weick, 1995) to interpret, understand, and respond to behaviors of their group members (Poole et al., 1989). Moreover, individuals may use GIFTs to make inferences about other followers within the same group (e.g., how similar or dissimilar when compared to the typical follower in the workgroup), which may influence how they think and behave (e.g., think and behave in accordance with the typical follower prototype).

Although individual members could vary in their conceptions of follower prototypes (i.e., IFTs), they also are likely to have shared conceptions of their group’s typical follower attributes (i.e., GIFTs) due to recurrent interaction and shared experiences as members of the same group (Klein and Kozlowski, 2000). The process by which group members internalize the key attributes of their groups (i.e., GIFTs) may be explained by the Social Identity model (e.g., Hogg, 2001; Hogg and van Knippenberg, 2003; van Knippenberg and Hogg, 2003; Giessner et al., 2009). To date, the Social identity model of leadership suggests that groups are represented by prototypical attributes and research show that the individual who best represents the group’s prototypicality is perceived as the leader of the group.

We propose a parallel notion whereby these prototypical attributes of the group also apply to followers, and followers who best embody these prototypical attributes are perceived as the typical or ideal followers. A fundamental assumption of the social identity model is that individuals perceive peer members by benchmarking the degree to which they match the attributes of the group’s leader and follower prototypes (Turner et al., 1987; Hogg, 2001). Given that members of the same workgroup are often exposed to the same information and experience, their leader and follower prototypes tend to be shared (Hogg and Terry, 2000). These shared leader and follower prototypes may influence how group members define themselves through the process of self-categorization (Turner et al., 1987). Such process is important as it determines whether a specific group member gets recognized as an in-group member or an out-group member, with individuals who best embody the group’s prototypical attributes categorized as in-group members while those who diverge from the prototype are classified as out-group members (Hogg and Terry, 2000).

Out-group members may be ostracized as deviants because they threaten the group’s collective identity. To avoid ostracism, group members may internalize prototypic attributes of GIFTs (e.g., incompetence) through the process of depersonalization (a process of self-stereotyping) because they want to be accepted by the group (Fenigstein, 1979) and avoid the detriments of being categorized as an out-group member (Hogg and Terry, 2000). For example, less competent peer members may shun and derogate a highly competent person as an overachiever and “know-it-all,” who violates prototypical norms for selfish gains at the expense of the group. To retain group membership and avoid ostracism, followers may act in accordance with and internalize the group’s follower prototype (e.g., scaling back effort and productivity). In short, group member’s personal (individual level) IFTs are likely to parallel that of GIFTs because recurrent interactions and shared experiences in the same group facilitate collective identification processes (i.e., “we” as opposed to “I”) (Tajfel and Turner, 1979; Turner, 1985). Moreover, even when individual-level IFTs conflicts with GIFTs, group members are likely to conform to GIFTs rather than relying on their own personal IFTs to the extent that they self-identify with the group and desire to maintain membership (Hogg and Reid, 2006).

Group-level Implicit Followership Theories are expected to activate corresponding behaviors due to the perception-behavior link (Bargh et al., 1996; Chen and Bargh, 1997). Research has found a link between perceptions and behaviors because just as cognitive concepts are represented mentally, so are social behavioral responses, and one is likely to activate the other via spread activation (Collins and Loftus, 1975; Dijksterhuis and Van Knippenberg, 1998). Numerous types of related mental representations and behavioral patterns are triggered when GIFTs are activated. For instance, when negative GIFTs are activated, they trigger related mental representations (e.g., “bad followers” activates the associated notion of “inexperienced followers”) and behavioral patterns (e.g., low effort expenditure) which are consistent with the activated concepts. The activation of mental representations (i.e., GIFTs) increases the tendency for individuals to behave in ways that are consistent with those cognitions. Meaning, followers in groups with more negative GIFTs may think and behave more negatively than those in groups that hold less negative GIFTs (McGregor, 1960; Chen and Bargh, 1997). Hence, negative GIFTs are expected to serve as expectations for followers, triggering processes like Golem effects.

### Group’s Implicit Followership Theories and Self-Efficacy

Negative GIFTs are the negative conceptions that group members have of followers (Sy, 2010). Although there may be some differences in follower prototypes across workgroups, negative GIFTs have been shown empirically to be shared across workgroups and individuals (e.g., Sy, 2010). On the basis of the perception-behavior link, negative GIFTs should negatively influence how followers feel about their capabilities, generating outcomes like Golem effects (Chen and Bargh, 1997). Concepts relating to “how group’s follower prototypes are” (i.e., GIFTs) are highly related and correspond to “how prototypical I am.” Given that individuals may internalize and embody the group’s negative follower prototypes via the Social Identity model explained above (Turner et al., 1987; Hogg and Reid, 2006; Hornsey, 2008), individuals who assimilate more negative GIFTs should have lower self-efficacy. That is, followers who internalize more incompetency conceptions of followership may believe they lack capabilities to perform well. Therefore, followers in groups that are exposed to more negative conceptions of followers would likely have lower self-efficacy. Accordingly, we hypothesize that:

**H1.** Negative group’s implicit followership theories (GIFTs) are negatively associated with followers’ self-efficacy.

### Self-Efficacy and Effort

We expect that followers’ self-efficacy will be positively related to the amount of effort they put forth. According to perception-behavior link (Bargh et al., 1996; Chen and Bargh, 1997), individuals who see themselves as more capable of accomplishing tasks (i.e., high self-efficacy) may put forth more effort when they encounter challenges. On the contrary, individuals who think that they are incapable (i.e., low self-efficacy) are likely to abate their effort when faced with obstacles (Bandura, 2000). Indeed, conceptual and empirical evidence have shown support for the linkage between self-efficacy and effort. Researchers have suggested that self-efficacy is key in determining whether employees’ work will be initiated and how much effort will be put forth (e.g., Stajkovic and Luthans, 1998), which suggest that effort is an outcome of self-efficacy (Bandura, 1997). Moreover, other researchers have found a positive association between self-efficacy and effort, such that having higher self-efficacy encourages individuals to put forth their best effort (e.g., Crawford et al., 1980; Eden, 1990). As such, we hypothesize:

**H2.** Followers’ self-efficacy is positively related to their effort.

### Effort and Performance

We posit that followers’ effort is positively associated with their performance. Although there are multiple factors that may influence individuals’ performance, the most direct influence may stem from the individual—the amount of effort an individual is willing to put forth. As suggested by researchers, individuals’ effort is one of the most common factors in influencing performance (e.g., Brown and Leigh, 1996). Moreover, numerous studies have found a positive relationship between effort and performance (Katerberg and Blau, 1983; Gardner et al., 1989; Schermerhorn et al., 1990; Brockner et al., 1992; Blau, 1993). For instance, Katerberg and Blau (1983) found that the amount of effort real estate agents put forth was related to their sales, the number of listings, and commissions. Furthermore, Blau (1993) found that bankers’ effort was associated with their overall performance. Altogether, these studies provide strong support for the positive relationship between individuals’ effort and performance. As such, we hypothesized that:

**H3.** Followers’ effort is positively associated with their performance.

### Multilevel Mediation Through Self-Efficacy and Effort

Our model for Golem effects (see **Figure 1**) and the above hypotheses suggest a mediation effect. Consistent with our prior propositions with the Social Identity model (Hogg, 2001; Hogg and van Knippenberg, 2003; van Knippenberg and Hogg, 2003) and perception-behavior link (Bargh et al., 1996; Chen and Bargh, 1997), we expect that group’s follower prototypes (i.e., negative GIFTs) would serve as a proxy for performance expectation influencing followers’ self-efficacy which in turn impact their effort and overall performance. Our model for Golem effects is labeled as a 2-1-1-1 model in which the influence of a level-2 variable (i.e., negative GIFTs) on a level-1 variable (i.e., followers’ performance) is conveyed by a sequence of two level-1 variables (i.e., followers’ self-efficacy and effort) (Krull and MacKinnon, 2001). Accordingly, we hypothesize:

**H4.** Negative group’s implicit followership theories (negative GIFTs) are significantly and indirectly related to followers’ performance through followers’ self-efficacy and effort.

## Materials and Methods

### Sample and Data Collection Procedure

A team of trained undergraduate research assistants recruited adult workgroups from their existing network of contacts. Survey data were collected from a wide range of industries (e.g., sales, entertainment, and healthcare). All workgroups consisted of one supervisor and two of his or her direct subordinates. Research assistants received approximately 1-h of training before the data collection process. Training included discussions of ethical guidelines for the recruiting procedure (e.g., no coercion) and qualifications (e.g., working adult). The workgroup leaders and followers completed different versions of the survey. The follower variables (self-efficacy and effort) were self-reported by followers, whereas the followers’ performance was assessed by the workgroup leaders. Both leaders and followers rated the group level variable (i.e., GIFTs). Followers’ self-reporting on self-efficacy and effort is appropriate because these variables represent individuals’ intrapsychic phenomena (Harris and Rosenthal, 1985; Sy, 2010). Hence, it would be more valid to ask followers about their perceptions and behaviors rather than observers who may lack the precision in judging followers. However, this approach may raise the concern of common method bias (Podsakoff et al., 2003). Such concern will be addressed below using different approaches to show that the same source ratings did not significantly bias the results.

The sample consisted of 303 participants: 101 workgroup leaders and 202 workgroup followers. Regarding workgroup followers, 58.4% were female, and 41.6% were male with mean age of 28.52 years (*SD* = 11.23). Followers were ethnically diverse, including Asians (34.2%), Hispanic/Latinos (25.7%), Caucasians (18.8%), African Americans (4.5%), Native Hawaiian/Pacific Islanders (0.5%), and some that were identified as “others” (16.3%). Regarding leaders (*n* = 101), 58.4% were female and 41.6% were male with mean age of 29.55 years (*SD* = 11.82). Leaders are also ethnically diverse, which includes: Asians (39.6%), Hispanic/Latinos (26.7%), Caucasians (13.9%), African Americans (3.0%), Native Hawaiian/Pacific Islanders (3.0%), and some that were identified as “others” (13.9%).

## Measures

### Group Members’ Implicit Followership Theories (GIFTs)

Group members’ negative conceptions of followers (GIFTs) were adapted and assessed using three negative attributes from the IFTs scale (Sy, 2010). Although implicit measures are rarely used in organizational research, a projective method was used to assess GIFTs in the current study because implicit measures tend to yield more reliable psychological construct in some instances (Roberts et al., 2006). For example, such approach allows us to capture individuals’ GIFTs while avoiding potential issues with the self-report method such as socially desirable responding (Harms and Luthans, 2012; Epitropaki et al., 2013). To assess GIFTs using the projective method, participants were provided the following instructions:

“In the following, you will see three statements describing a story. Imagine the typical members/followers in your workgroup in these stories. Your task is to invent stories for the typical members/followers in your workgroup. Please write a short story in the space provided…think about what led to this event, what is happening now, and the outcomes at the end…there are no right or wrong stories. Imagine whatever kind of story you like.”

Based on the instructions, participants then were asked to invent stories about typical members/followers of their workgroup in typical scenarios at work (e.g., group member’s daily experience at work). For example, one participant wrote:

“[Group member] showed up late to work, was already feeling stressed out from problems at home and is overwhelmed but decides to carry on anyways, attempting to act as if nothing is happening. Suddenly a timed order comes through the work system which he feels unable to accomplish on his own. He manages to complete his task at hand even though it did take him longer than expected. [Group member]’s supervisor reprimands him for not being able to finish the task in a timely manner like a manager should and because of such lack in performance has his schedule altered to reflect his supervisor’s distrust in his abilities. Such action leads [group member] to feel unappreciated for his effort in his work environment which causes him to care less and less about his involvement and overall progress at work, leaving him frustrated even after leaving the workplace.”

Using the IFTs scale, they were then asked to indicate on a 7-point scale how accurate each item described the typical group members in the stories. The IFTs scale comprised of three negative dimensions, each consisting three items. Such method allows leaders and followers to describe and assess typical member in their workgroups based on specific work-related scenarios (versus less relevant social functions). For investigating Golem effects (i.e., negative self-fulfilling processes) in this study, it is appropriate for us to focus on the negative dimensions of GIFTs. Specifically, we focus on the Incompetency dimension (i.e., uneducated, slow, inexperienced) because it is directly related to individuals’ performance. The Incompetency dimension was constructed by aggregating the three items (i.e., uneducated, slow, inexperienced). The internal consistency coefficient for negative GIFTs was 0.89.

We conceptualize GIFTs as a compositional emergent construct of IFTs because they are measured by the same set of items and are structurally and functionally equivalent (Klein and Kozlowski, 2000). Composition emergence is germane to phenomena that progress through recurrent within-group interactions in which core group elements (i.e., cognition, perception) become shared among all members within a group (Kozlowski and Chao, 2012). Similarly, as group members interact, their conceptions of the characteristics that reflect the group’s prototypical follower (i.e., GIFTs) should converge and become shared over time. Therefore, it is appropriate for us to conceptualize GIFTs as a compositional emergent construct and calculate a single score for each workgroup.

Before calculating a single score for each workgroup by aggregating group members’ ratings, we conducted analyses to justify whether there is sufficient support for both within-group agreement and between-group variation (Klein et al., 1994). Specifically, we followed Klein and Kozlowski’s (2000) recommendations to account for group-level analysis as a shared team-construct. First, we conducted a one-way analysis of variance (ANOVA) to estimate between-group variability. Results suggested significant group effects on individuals’ ratings of negative GIFTs: *F* (100, 202) = 2.01, *p* < 0.01 (*ICC*_1_ = 0.25 and *ICC*_2_ = 0.50). In addition, we calculated *r_wg(j)_* statistics to assess the extent of within-group agreement for negative GIFTs (Biemann et al., 2012). The median within-group agreement value for negative GIFTs was considered strong (Negative GIFTs: *r_wg(j)_* = 0.84; James et al., 1984; Bliese, 2000; LeBreton and Senter, 2008). All in all, our analyses revealed that our shared group construct, negative GIFTs, have both between-group variability and within-group homogeneity.

### Self-Efficacy

We used five items adapted from Riggs and Knight’s (1994) measure of self-efficacy to assess followers’ self-efficacy. Workgroup followers were asked to respond to five items on a 7-point scale regarding their own self-efficacy. Example items included, “I have confidence in my ability to do my job” and “I have all the skills needed to perform my job very well.” The internal consistency coefficient for the scale was 0.70.

### Effort

Workgroup followers’ effort was measured with five items adapted from Brown and Leigh (1996). Followers were asked to rate on a six-point scale the extent to which each statement describe themselves. Example items included, “I strive as hard as I can to be successful in my work.” and “When I work, I really exert myself to the fullest.” The internal consistency coefficient for the scale was 0.94.

### Work Performance

Leaders of each workgroup rated their followers’ work performance. Followers’ work performance was measured with three items using a 7-point scale that adapted from Wayne et al. (1997). Example items included, “This employee has performed his/her job well,” and “In my estimation, this employee gets his/her work done very effectively.” The internal consistency coefficient for the performance was 0.90.

### Controls

We controlled for participants’ age as it may influence performance (e.g., Blanchard-Fields et al., 2007), and because IFTs may continue to be refined and further developed over time as individuals interact with others (Lord and Maher, 1991; Sy, 2010). We also included gender as a second control variable (1 = male, 2 = female) because previous studies have found self-fulfilling prophecies to be more potent with men (McNatt, 2000). Finally, we controlled for participants’ average hours worked per week because performance can be a function of effort and time spent practicing one’s craft (Yeo and Neal, 2004).

## Results

### Analysis

To accommodate the multi-level nature of the study, we used multilevel structural equation modeling (MSEM) to model top-down (2–1) relationships (Preacher et al., 2010). The MSEM models dismantle the variance of a variable into its latent within-unit variance and a latent between unit variance (Lüdtke et al., 2008). By dismantling variance into components at the between and within levels, MSEM avoids potential problems of conflated within and between level relationships in traditional multi-level approach (e.g., hierarchical linear modeling), allowing us to estimate indirect relationships more precisely (Zhang et al., 2009; Preacher et al., 2010).

Hypotheses 1 through 4 suggests an indirect relationship in which negative GIFTs and followers’ performance are mediated by followers’ self-efficacy and effort. Using MSEM, we could simultaneously evaluate the top-down relationships between (a) negative GIFTs and followers’ self-efficacy, (b) the individual-level relationship between followers’ self-efficacy and effort both within- and between-group, (c) the individual-level relationship between followers’ effort and performance both within- and between-group. The indirect relationship between negative GIFTs and followers’ performance mediated by followers’ self-efficacy and effort (Hypothesis 4) was tested using the product-of-coefficients methods. Preacher et al. (2010) suggested that a level-2 variable’s top-down relationship with a level-1 outcome is a between-group relationship because the level-2 variable could not predict the within-group variances among individuals in the workgroups. Therefore, we examined the coefficient for level 2 predictor (negative GIFTs) and the latent group mean of level-1 outcome (followers’ performance).

The means, standard deviations, reliabilities and correlations among the study variables are presented in **Table 1**. All analyses were conducted using Mplus 7.0 (Muthén and Muthén, 2012–2017) with robust maximum likelihood (MLR) estimation. Model fit for the 2-1-1-1 multi-level mediation was assessed using the root-mean-square error of approximation (RMSEA), the Tucker-Lewis Index (TLI), and the comparative fit index (CFI) (Hu and Bentler, 1999). To compare multilevel models, the scaled chi-square difference test (Satorra, 2000) was used for comparisons.

**Table 1:** Means, standard deviations, reliability coefficients and correlations.

Variable	*M*	*SD*	1	2	3	4	5	6	7
1. Negative GIFTs	2.39	0.84	(0.89)						
2. Self-efficacy	5.97	0.69	−0.35**	(0.70)					
3. Effort	5.97	1.09	−0.16*	0.39**	(0.94)				
4. Performance	6.09	1.06	−0.24**	0.15**	0.24**	(0.91)			
5. Gender	1.58	0.49	−0.04	0.04	−0.01	0.09	–		
6. Age	28.52	11.23	−0.03	0.10	0.17*	0.03	−0.11	–	
7. Hours per week	27.37	13.84	−0.09	0.16*	0.19*	−0.03	−0.17	0.49**	–


*Note. Internal consistency reliabilities are on the diagonal in parentheses*.

^∗∗^*p* < *0.01*. ^∗^*p* < *0.05*.

### Discriminant Validity of Constructs

Prior to testing the hypotheses, we conducted confirmatory factor analyses to examine the distinctiveness of the study variables. Original items were used as indicators for all measures. For the hypothesized four-factor model, results indicated that all factor loadings were significant (*p* < 0.001). Standardized factor loadings were on average 0.86 for negative GIFTs, 0.60 for self-efficacy, 0.87 for effort, and 0.88 for performance. In addition, results indicated that the model fit for our hypothesized four-factor model is considered acceptable (χ^2^ (98) = 262.49, *N* = 202; CFI = 0.92, TLI = 0.91, RMSEA = 0.09) and is better than a one-factor model (Δχ^2^ (6) = 880.51, *N* = 202; CFI = 0.51, TLI = 0.44, RMSEA = 0.22) as well as a model in which variables are loaded into three factors based on raters (Δχ^2^ (3) = 108.63, *N* = 202; CFI = 0.87, TLI = 0.85, RMSEA = 0.12). (Hu and Bentler, 1999). Hence, given the high factor loadings and the greater fit of the hypothesized four-factor mode, we conclude that our measures captured distinct constructs.

### Common Method Variance

As mentioned in the previous section, both leaders and followers were used to avoid biases from same source ratings. However, common method variance (CMV; i.e., variance as a product of the measurement method rather than the constructs of the measures) may have biased the some of the variables measured from followers’ perceptions (i.e., GIFTs, self-efficacy, effort) (Podsakoff et al., 2003). To ensure CMV did not significantly bias the results of the study, we conducted a confirmatory factor analysis to assess whether a single-factor accounted for most of the covariance between the study variables. If CMV is responsible for the relationship among variables, the single-factor CFA would fit the data well (Korsgaard and Roberson, 1995; Mossholder et al., 1998). The results suggested that a single-factor model with followers’ same source ratings as indicators was significantly worse fitting compared to the hypothesized model (χ^2^ (*N* = 202, 65) = 671.51, *p* < 0.01; CFI = 0.63; TLI = 0.56; RMSEA = 0.22), suggesting that CMV did not bias the results of our study.

### Hypothesis Testing

The multilevel structural model, in which negative GIFTs and followers’ performance are associated through followers’ self-efficacy and effort, showed a good fit overall (χ^2^ (4) = 1.68, *p* = 0.79, CFI = 1.00, TLI = 1.28, and RMSEA = 0.00). All factor loadings were significant (ps < 0.01). We tested an alternative model with a direct path from negative GIFTs to followers’ performance. The added pathway did not improve the overall model fit (χ^2^ (3) = 1.71, *p* = 0.63, CFI = 1.00, TLI = 1.21, and RMSEA = 0.00) as indicated by a non-significant scaled chi-square difference test (Δχ^2^_scaled_ (1) = 0.06, *p* = ns), which serves as evidence for a full mediation. Hence, we retain the hypothesized model.

Next, we examined the results of all direct and indirect relationship in our model; shown in **Table 2**. First, negative GIFTs were negatively related to followers’ self-efficacy, as indicated by a significant unstandardized structural coefficient (*b* = −0.27, *p* < 0.01), which supports Hypothesis 1. Followers’ self-efficacy was significantly related to followers’ effort at both the within-level of analysis (*b* = 0.45, *p* < 0.01) and between-level of analysis (*b* = 0.78, *p* < 0.01), supporting Hypothesis 2. Followers’ effort was found to be positively related to followers’ performance as indicated by an unstandardized structural coefficient in between-level of analysis (*b* = 0.99, *p* < 0.01); however, the relationship at the within-level analysis was insignificant (*b* = −0.11, *p* = 0.07). This result suggested that individuals’ effort was not related to their performance; however, groups that exhibit more effort tend to perform better at work. This result provided partial support for Hypothesis 3. As for the multi-level mediation model, negative GIFTs had a negative and statistically significant indirect relationship with follower performance, through followers’ self-efficacy and effort (unstandardized estimate of the product of coefficients = −0.21, *p* < 0.05, 95% CI = −0.37, −0.05), supporting the mediation in Hypothesis 4.

**Table 2:** Tests of direct and indirect relationships (Hypotheses 1–4)

Path	Estimate	S.E.	Lower and upper 95% CI limits
**Test of direct relationships**			
*Top-down direct path (2–1)*			
Negative GIFTs → self-efficacy (Hypothesis 1)	−0.27**	0.06	(−0.37, −0.17)
**Direct paths (1–1)**			
*Self-efficacy → effort (Hypothesis 2)*			
Within-level relationship	0.45**	0.11	(0.28, 0.63)
Between-level relationship	0.78**	0.20	(0.46, 1.10)
*Effort → performance (Hypothesis 3)*			
Within-level relationship	−0.11	0.06	(−0.21, −0.01)
Between-level relationship	0.99**	0.35	(0.42, 1.56)
**Test of indirect relationships**			
*Indirect paths model (2-1-1-1)*			
Negative GIFTs → self-efficacy → effort → performance (Hypothesis 4)	−0.21*	0.10	(−0.37, −0.05)


*Note. Unstandardized estimates are reported for both direct and indirect relationships*.

*1 = level-1 variable 2 = level-2 variables; CI = confidence interval*.

^∗∗^*p* < *0.01*. ^∗^*p* < *0.05*.

## Discussion

In this study, we examine the relationship between negative GIFTs and naturally occurring Golem effects. Specifically, we test whether negative GIFTs are associated with followers’ performance through their self-efficacy and effort. GIFTs reflect the prototypical follower attributes for the group, from which followers make inferences about themselves as well as other followers. Negative GIFTs are the in-group standard for followers, which serve as negative expectations for followers to fulfill to be accepted and avoid ostracism as an out-group member (Hogg and Terry, 2000). Followers who internalize more negative prototypical follower attributes of the group viewed themselves as less capable. This, in, turn, trigger Golem effects. We find support for the hypothesized multi-level model as shown in **Figure 1**. In line with our hypotheses, we find a negative top-down relationship between negative GIFTs and followers’ self-efficacy. Furthermore, followers’ self-efficacy is positively related to their effort, which is related to their overall performance. Additionally, we find a negative indirect relationship between negative GIFTs and follower performance through follower self-efficacy and effort.

Our results indicate that followers’ effort and performance are positively related only at the between-level of analysis. Interestingly, although this relationship at the within-level is insignificant, the marginally negative relationship between effort and performance warrants some discussion. One possibility for these results may be due to social loafing (Latané et al., 1979). While each employee may take on different roles on a team, they are likely to be working collectively on tasks or projects rather than individually. As such, it is possible that followers exert less effort while working in groups. This is supported by previous research. For example, Kerr and Bruun (1983) found that individuals working in teams tend to exert less effort when a certain performance level is reached, which may have been influenced by the norms of negative GIFTs; Individuals tend to match their group members’ effort while working collectively (Jackson and Harkins, 1985). Moreover, those who attempt to exert more effort may be evaluated by their supervisors more negatively for violating norms associated with GIFTs (i.e., extra effort viewed as a selfish attempt to overachieve at the expense of less competent peer members). Though speculative, these results suggest that GIFTs serve as expectations that are enforced via social identity processes regardless if they are positive or negative in nature.

### Implications

Our findings have several significant implications. First, this study contributes to the self-fulfilling prophecies literature by providing the first empirical evidence for naturally occurring Golem at work. Moreover, we offer a solution for investigating Golem effects via IFTs that circumvents the ethical concerns that have hampered research for decades. As Eden (1990) noted, individuals’ expectations occur naturally, so this approach captures the phenomenon in its most ecologically valid context. Second, our study further develops the empirical work on IFTs and extends what has been viewed as individual level constructs (IFTs; Sy, 2010; Epitropaki et al., 2013) to group level constructs as viewed from the lens of the social identity approach model (self-categorization theory; Turner, 1985). Specifically, we examine whether GIFTs (group level construct) is associated with followers’ self-efficacy, effort, and performance (individual level constructs). Our model takes the multi-level nature of workgroups into account and provides a more accurate estimate for both within-and between-level of analysis (Zhang et al., 2009; Preacher et al., 2010). Third, while researchers have examined leadership processes via the social identity model (Hogg, 2001; Hogg and van Knippenberg, 2003; van Knippenberg and Hogg, 2003), we advance a parallel model for followership processes that provide further insights and addresses gaps in the followership literature. Indeed, a rich body of research has accumulated around leaders and leadership process (Yukl, 2012), whereas research on followership process is scant (Carsten et al., 2010; Sy, 2010; Bligh et al., 2011). Advancing our understanding of followership is essential given that leadership can only occur if there is followership; there can be no leaders if there are no followers (Uhl-Bien et al., 2014). Hence, we appropriately examine the process of followership within a group context and how such a process may influence followers’ overall performance. Lastly, as we bring to light that Golem effects occur naturally, it may benefit organizations to assess whether GIFTs are a factor affecting group performance. Organizations may intervene to restrain Golem effects by creating more positive expectations about group members. For example, group members may engage in a writing intervention describing their ideal or “best possible member” (Layous et al., 2013). In line with our prior propositions, this intervention may create more positive expectancies by redefining the salient attributes of group identity by which members self-identify and internalize (Turner et al., 1987; Hogg and Reid, 2006).

### Limitations

Despite its contributions and methodological strength such as the use of MSEM, this study, like any other studies, is not without its limitations. First, the cross-sectional nature of the study design prevents us from demonstrating causal directions. However, this design is a necessity for examining naturally occurring (i.e., without any form of artificial manipulation) Golem effects at work. Moreover, our approach offers an appropriate solution to circumvent the ethical concerns mentioned previously. Second, although the direction of relationships in our model is derived theoretically, followers’ performance could also influence groups’ conceptions of followers. Meaning, negative GIFTs could also be an outcome variable rather than a predictor variable (i.e., a recursive loop from followers’ performance to negative GIFTs). Lastly, it is possible that the duration group members spent working together may impact how their GIFTs are formed because individuals’ GIFTs may change based on their interactions with other group members (Lord and Maher, 1991). Hence, researchers may consider investigating how GIFTs emerged and transformed by implementing a longitudinal study design (Kozlowski and Chao, 2012).

### Future Research Directions

Future research should investigate the impact of negative LIFTs (Leader’s IFTs) on Golem effects and followers’ performance (Babad et al., 1982). While research has demonstrated the positive interpersonal expectancy effect (Naturally occurring Pygmalion effects; Whiteley et al., 2012), studies have yet to investigate this negative interpersonal expectancy effect. Aligned with the claim of our study and the literature on self-fulfilling prophecies (Eden, 2003), leaders with more negative conceptions of followers (i.e., negative LIFTs) should have more negative expectations of their followers which may trigger Golem effects, impairing followers’ performance. As mentioned previously, research on Golem effects is often bounded by their inability to manipulate negative expectations due to ethical concerns. Using negative LIFTs, however, allow researchers to investigate interpersonal Golem effects while avoiding these ethical concerns.

The current study focused on the Incompetence dimension of negative GIFTs because we aimed to examine the traditional framework of Golem effects (i.e., Incompetence is most relevant to performance expectations). However, researchers may investigate an expanded theory of Golem effects using the Insubordinate and Conformity dimensions of GIFTs. For instance, how might the Insubordination dimension influence relationships among group members? Followers who internalize more insubordinate attributes may engage in more adverse behaviors (e.g., followers may be arrogant and mistreat members of the group) because they assume these behaviors are normative. This, in turn, may damage the relationships among members that cause detrimental outcomes. Good relationships lead to positive outcomes, whereas bad relationships have the opposite effect. For example, leader-follower dyads that have good relationships often lead to positive work outcomes, such as higher job satisfaction, job commitment, and organizational citizenship behaviors (Gerstner and Day, 1997; Ilies et al., 2007; Dulebohn et al., 2012). Thus, we propose an expansion of Golem effects by suggesting that it may operate via other mediators (e.g., relationship quality) beyond its core variables (i.e., efficacy and effort).

In addition, it might be fruitful to examine the outcomes related to the Conformity dimension of GIFTs. Although conformity is often viewed as a negative attribute in Western cultures, it may be a positive feature for followers in other cultures that endorse different types of follower attributes (Epitropaki et al., 2013). Indeed, researchers have found that individuals have different expectations for followers in Eastern and the Western cultures (Sy et al., 2017). Whereas followers in Western cultures are expected to take on a more proactive role (e.g., make suggestions and speak up in meetings), followers in Eastern Cultures tend to conform to their leaders as a respectful gesture (e.g., execute tasks without questioning their leaders, and remain silent during meetings). As such, investigating the Conformity dimension using an Eastern cultural sample may lead to the opposing predictions compared to what is expected in Western cultures—planting Galatea effects in Eastern soil with a Golem seed.

Lastly, future studies on GIFTs may emulate the multi-level structural equation method and investigate how a level three variable (e.g., culture or organizational structure) may transform GIFTs. Organizational culture may be a source of alignment or discrepancy for IFTs at the group and individual levels (Fitzsimons et al., 2008; Sy, 2010). In addition, GIFTs that are endorsed by companies with traditional hierarchical structures may differ from those with flat or horizontal structures.

## Conclusion

Self-fulfilling prophecies reflect a double-edged sword (Eden, 1990). While positive expectations may promote positive outcomes, negative expectations can lead to detrimental outcomes. Much knowledge has accumulated on positive expectancy effects. In contrast, we know little about the dark side of self-fulfilling prophecies. There is still much to be learned about Golem effects. This study is a first step toward understanding how GIFTs may play a key role in summoning the detrimental consequences of Golem effects. It is important to note that even the most productive and gifted employees may be restrained when operating in workgroups that have high negative GIFTs. All in all, insights gained from GIFTs research may allow researchers to understand how Golem effects may be restrained so that employees may unleash their talents and transform as positive gifts of group performance.

## Ethics Statement

This study was conducted in accordance with the ethical guidelines set by the Institutional Review Board of the University of California, Riverside with written informed consent from all subjects. Employees participated in our study voluntarily. The protocol was approved by the Institutional Review Board committee of the University of California, Riverside.

## Author Contributions

AL and TS designed the study and collected the data. AL analyzed the data and drafted the paper while TS revised/edited the paper.

## Conflict of Interest Statement

The authors declare that the research was conducted in the absence of any commercial or financial relationships that could be construed as a potential conflict of interest.
